# Contribution of the *-160C/A* Polymorphism in the *E-cadherin* Promoter to Cancer Risk: A Meta-Analysis of 47 Case-Control Studies

**DOI:** 10.1371/journal.pone.0040219

**Published:** 2012-07-05

**Authors:** Lin Wang, Guiying Wang, Chenqi Lu, Bo Feng, Jiuhong Kang

**Affiliations:** 1 Endocrinology Department, Shanghai East Hospital, Shanghai Key Laboratory of Signaling and Disease Research, School of Life Science and Technology, Tongji University, Shanghai, People’s Republic of China; 2 Laboratory of Population and Quantitative Genetics, Institute of Biostatistics, SKLGE, School of Life Sciences, Fudan University, Shanghai, People’s Republic of China; Dartmouth Medical School, United States of America

## Abstract

**Background:**

The *-160C/A* polymorphism (rs16260) of *E-cadherin*, a tumor repressor gene, has been shown to be a tumor susceptibility allele for various types of cancers. Because the significance of this polymorphism to cancer risk has been recognized, there are increasing studies investigating *-160C/A* in different types of cancers and ethnic populations. However, there is still uncertainty about the level of risk for a variety of cancers.

**Methods:**

To resolve the controversial question raised by these studies as of March 2012 and provide more statistical power for detecting the significance of *-160C/A*, we performed a meta-analysis of 47 case-control studies in 16 types of cancers (18,194 cases and 20,207 controls). A meta-regression model and subgroup analysis were employed to identify the source of heterogeneity. Publication bias was evaluated, and sensitivity analysis and cumulative evidence assessment were also performed.

**Results:**

Using fixed- and random-effects models, the *-160AA* homozygote was more susceptible to urothelial cancer compared with the *-160CA* heterozygote. Additionally, the *-160A* allele is an ethnicity-dependent risk factor for prostate and colorectal cancers. Carriers of the *-160A* allele in Asians and Europeans were more susceptible to prostate cancer, whereas their North American counterparts seemed tolerant. The *-160AA* homozygote plays a protective role for Europeans who develop colorectal cancer. The stability of these observations was confirmed by a one-way sensitivity analysis. However, the cumulative evidence for all cancer types was considered ‘weak’ using the Venice guidelines.

**Conclusions:**

A meta-analysis indicated that the *-160A* allele of *E-cadherin* provides a higher risk for the development of prostate and urothelial cancers and a protective role for colorectal cancer in an ethnicity-dependent manner.

## Introduction

*E-cadherin*, which has a widely acknowledged role in cell-cell adhesion, also functions as an invasion/tumor suppressor gene. Several immunohistochemical studies have reported a strong correlation between E-cadherin loss and the occurrence of tumors. The downregulation of E-cadherin is generally due to transcriptional repression [1]. The *-160C/A* polymorphism in the promoter region of the *E-cadherin* gene has been reported to have a direct effect on its transcriptional regulation and therefore may influence susceptibility to cancers [2]. To identify whether the *-160C/A* polymorphism of *E-cadherin* is involved in the pathogenesis of tumors *in vivo*, case-control studies concerning this allelic variation and cancer risk have been broadly performed. However, there is still uncertainty about the level of risk for a variety of cancers in a number of studies investigating the effect of *-160C/A* on different types of cancers and ethnic populations.

To resolve the controversial question raised by this evidence and provide more statistical power for detecting the significance of *-160C/A* to cancer risk, we performed a meta-analysis on the *160C/A* polymorphism of *E-cadherin* and cancer risk with 47 case-control studies including 18,194 cases and 20,207 controls as of March 2012. The results indicated that the *-160A* allele of *E-cadherin* leads to a higher risk for the development of prostate and urothelial cancers and is an ethnicity-dependent risk factor for prostate and colorectal cancers. The significance of the *-160C/A* polymorphism in developing various types of cancer has received increasing attention. However, further observation will be needed to improve the evaluation power of association.

## Methods

### Search Strategy

We conducted a systematic literature search using the databases MEDLINE (US National Library of Medicine, Bethesda, Maryland) and PubMed (National Center for Biotechnology, National Library of Medicine) as of March 2012 with the keywords “polymorphism of the *E-cadherin* gene,” “rs16260,” and “-160C/A,” in combination with “cancer,” “tumor,” “neoplasm” or “carcinoma.” The full texts of the candidate articles were carefully examined for data extraction, and the reference lists were also reviewed to identify further relevant studies for our previous report [3].

### Inclusion Criteria

Case-control studies with sufficient published data for estimating an odds ratio (OR) and corresponding 95 percent confidence interval (95% CI) were included in this meta-analysis. Published meta-analyses on the association of polymorphisms of *E-cadherin* with cancer risk were included in the assessment of evidence.

### Data Extraction

The following information was independently extracted from each study by two investigators: 1) publication date, first author, year of publication, and country of origin; 2) polymorphism of the *E-cadherin* gene and cancer types; 3) characteristics of cases and controls and genotyping method; and 4) number of cases and controls with heterozygous and homozygous genotypes. This information is summarized in Tables 1, S1 and S2.

**Table 1:** Estimates of odds ratios and the corresponding 95% confidence intervals for *AA* and *CA* genotype and *A* allele carriers versus the *CC* genotype for 16 types of cancers analyzed by fixed- or random- effects models divided by cancer type and ethnicity as of March 2012.

Cancer type	No. ofdata set	No. of cases	No. of controls	*AA*		*CA*		*(AA+CA)*	
				OR^†^	95% CI	OR	95% CI	OR	95% CI
Gastric	19	3,453	4,775	1.14	0.85, 1.52	1.01	0.88, 1.16	1.03	0.90, 1.18
Asian#	11	2,164	2,558	0.96	0.63, 1.46	0.92	0.78, 1.07	0.93	0.80, 1.08
European#	6	1,102	2,046	1.15	0.78, 1.69	1.18	0.89, 1.57	1.20	0.91, 1.58
Others#	2	187	171	2.95	0.90, 9.69	1.36	0.86, 2.14	1.53	0.99, 2.36
Healthy⋇20;	11	1,929	2,100	1.19	0.84, 1.70	0.93	0.76, 1.15	0.97	0.80, 1.18
Healthy matched⋇20;	3	356	367	1.14	0.14, 9.35	1.14	0.70, 1.84	1.20	0.62, 2.32
CAG⋇20;	1	96	196	1.45	0.45, 4.64	1.36	0.81, 2.27	1.37	0.83, 2.24
Free of cancer⋇20;	4	1,072	2,112	1.10	0.80, 1.52	1.06	0.88, 1.28	1.05	0.90, 1.18
Colorectal	9	7,117	7,157	0.85	0.71, 1.03	0.97	0.86, 1.08	0.95	0.85, 1.05
Asian#	2	356	294	0.90	0.03, 25.97	1.22	0.84, 1.75	1.20	0.84, 1.73
European#	7	6,761	6,863	0.85	0.74, 0.99	0.95	0.84, 1.07	0.93	0.83, 1.03
Healthy⋇20;	5	6,325	5,877	0.82	0.63, 1.06	0.94	0.84, 1.05	0.91	0.84, 0.99
Free of CRC⋇20;	3	686	1,034	0.85	0.58, 1.26	0.88	0.62, 1.26	0.86	0.60, 1.23
Free of cancer⋇20;	1	106	246	1.49	0.64, 3.43	1.32	0.82, 2.14	1.35	0.85, 2.13
Esophageal	2	407	490	1.03	0.27, 3.93	1.30	0.97, 1.73	1.22	0.93, 1.61
Prostate	10	3,570	3,304	1.36	0.93, 1.99	1.32	1.11, 1.58	1.33	1.11, 1.60
Asian#	3	655	726	1.85	0.98, 3.50	1.51	1.14, 2.00	1.56	1.16, 2.08
European#	5	2,251	2,106	1.31	0.83, 2.07	1.34	1.00, 1.80	1.35	1.02, 1.80
Others#	2	664	472	1.12	0.21, 5.88	1.12	0.87, 1.45	1.10	0.86, 1.41
Healthy⋇20;	2	974	646	0.69	0.45, 1.06	1.12	0.90, 1.38	1.02	0.84, 1.25
Healthy matched⋇20;	4	1,895	1,765	1.65	0.90, 3.02	1.18	1.02, 1.36	1.20	1.05, 1.37
Healthy and BPH⋇20;	2	419	546	1.85	1.05, 3.28	1.21	0.92, 1.60	1.29	0.99, 1.69
BPH⋇20;	1	200	159	6.21	0.74, 52.37	2.10	1.32, 3.33	2.20	1.40, 3.47
BPH and other⋇20;	1	82	188	1.65	0.41, 6.62	3.83	2.14, 6.85	3.60	2.03, 6.38
Urothelial	5	1,064	1,124	2.58	1.40, 4.76	1.54	0.99, 2.40	1.70	1.11, 2.61
Asian#	3	544	474	4.05	2.49, 6.60	1.82	0.86, 3.87	2.41	0.97, 5.99
European#	2	520	650	1.43	0.88, 2.34	1.17	0.91, 1.49	1.21	0.96, 1.53
Breast	4	1,142	1,063	1.14	0.83, 1.57	1.14	0.95, 1.37	1.14	0.96, 1.36
Pancreatic	1	254	101	2.52	1.21, 5.26	1.37	0.99, 1.88	1.62	1.00, 2.63
Nasopharyngeal	1	162	140	3.84	1.04, 14.15	1.81	1.04, 3.15	2.02	1.20, 3.41
Endometrial	1	92	246	1.25	0.46, 3.38	2.07	1.25, 3.42	1.93	1.19, 3.14
Cervical	1	101	246	2.08	0.96, 4.48	1.05	0.63, 1.74	1.22	0.77, 1.95
Ovarian	1	207	256	0.69	0.20, 2.40	0.95	0.64, 1.42	0.93	0.63, 1.37
Lung	1	95	85	12.56	0.68, 231.61	2.37	1.13, 4.99	2.81	1.36, 5.83
Oral	1	251	347	0.32	0.18, 0.57	0.66	0.47, 0.94	0.57	0.41, 0.80
Liver	1	131	347	0.77	0.42, 1.42	0.88	0.57, 1.36	0.85	0.56, 1.29
Thyroid	1	92	169	2.09	0.90, 4.87	2.42	1.39, 4.22	2.35	1.39, 3.99
Lymphoma	1	56	357	0.70	0.20, 2.47	0.94	0.52, 1.70	0.91	0.51, 1.60
Overall	59	18,194	20,207	1.21	1.03, 1.43	1.14	1.05, 1.23	1.16	1.07, 1.26

Statistically significant, with *P*<0.05 and a 95% confidence interval (CI) that does not include 1.0.

† OR, odds ratio.

# Stratified by ethnicity, including Asian, European, and others (North American and African).

⋇20;Stratified by controls, including benign prostatic hyperplasia (BPH), BPH or visitors or requesting vasectomy (BPH and others), benign urological patients matched, chronic atrophic gastritis (CAG), free of colorectal cancer (free of CRC), free of cancer, healthy, healthy and BPH, healthy and free of cancer, healthy matched, and normal peritumoral tissues.

### Meta-analysis

Based on the inclusion criteria, 47 case-control studies were included. In total, 59 datasets were extracted based on the original data, which were divided by either region or cancer type. Relevant information on the studies is summarized in Table S1. The review process and outcomes of inclusion and exclusion are illustrated in Figure S1.

Hardy-Weinberg equilibrium was tested in control samples of each dataset by the chi-square method to assess the latent bias resulting from the deviation of genotype distribution. ORs were considered as estimates of relative risk and were combined across studies using fixed- or random-effects meta-analysis for low and high heterogeneity, respectively. Heterogeneity was assessed using the *I^2^* statistic, which describes the degree of genuine differences across studies in a meta-analysis [4]. A meta-regression model was used to identify the source of heterogeneity [5], and subgroup analysis was also carried out. One-way sensitivity analysis was performed by removing one dataset at a time in cancer types containing more than three datasets [6], [7]. Publication bias was assessed using the modified test proposed by Harbord for small-study effects in meta-analyses of controlled trials with binary endpoints [8].

### Assessment of Cumulative Evidence

Venice interim guidelines were also introduced to assess the credibility of cumulative evidence, which evaluated the evidence using a semi-quantitative index that assigned three levels for the amount of evidence, extent of replication, and protection from bias [9], [10]. N_minor_ was the sum of *AA* homozygotes in cases and controls, and f _minor_ was the *A* allele frequency in control. Category A of amount required a sample size over 1,000 in the least common genetic group of interest; B corresponded to a sample size of 100–1,000, and C corresponded to a sample size of <100. Either one of the following situations corresponded to category C of replication: no association, no independent replication, or high heterogeneity (*I^2^*>50%). Nominal ORs (0.87–1.15) and significant bias detected by the Harbord test corresponded to category C of bias. Bias in data containing less than three data sets was considered as category B because there was no obvious bias in phenotype definition, genotyping, or population stratification according to the original document, but the Harbord test could not be carried out because of the paucity of data sets.

## Results

As of March 2012, there were a total of 47 case-control studies that included 18,194 cases and 20,207 controls in 16 types of cancers. Combined analysis of the extracted 59 datasets showed significant heterogeneity (*Q* = 177.76, *P*<0.00001, *I^2^* = 67%) among studies (Table 2, Figure S2). A meta-regression analysis was thus carried out to identify the source of heterogeneity, and three kinds of covariates were introduced, including cancer type, ethnicity and source of controls (Table 3). Meta-regression analysis revealed that when the source of controls was introduced in combination with cancer type, all of the heterogeneity could be adjusted in *-160A* carriers (*CA*, *I^2^* = 28%, adjusted *R^2^* = 100%, *P* = 0.003; *CA+AA*, *I^2^* = 33%, adjusted *R^2^* = 100%, *P* = 0.0003). The combination of ethnicity with either cancer type or source of controls primarily accounted for the heterogeneity in the *AA* homozygote (ethnicity and cancer type, *I^2^* = 46%, adjusted *R^2^* = 59%, *P* = 0.02; ethnicity, cancer type and source of control, *I^2^* = 45%, adjusted *R^2^* = 63%, *P* = 0.07). Furthermore, we carried out subgroup analysis according to the identified covariates (Table 2).

**Table 2:** Heterogeneity test for studies of each genotype in different cancer types (as of March 2012) with Cochrane’s *Q*-test and the quantity *I^2^*.

Cancer type	*AA*			*CA*			*(AA+CA)*			No. of data sets
	*Q* value	*P* value	*I^2^* (%)	*Q* value	*P* value	*I^2^* (%)	*Q* value	*P* value	*I^2^* (%)	
Gastric	34.62	0.01	48	30.98	0.03	42	33.44	0.01	46	19
Asian#	19.74	0.03	49	14.67	0.14	32	13.89	0.18	28	11
European#	7.86	0.16	36	12.62	0.03	60	13.29	0.02	62	6
Others#	2.01	0.16	50	0.15	0.70	0	0.83	0.36	0	2
Healthy⋇20;	15.62	0.11	36	19.59	0.03	49	19.71	0.03	49	11
Healthy matched⋇20;	16.95	0.00	88	4.32	0.12	54	8.50	0.01	77	3
CAG⋇20;	/	/	/	/	/	/	/	/	/	1
Free of cancer⋇20;	1.41	0.70	0	3.85	0.28	22	2.61	0.46	0	4
Colorectal	10.06	0.26	20	12.28	0.14	35	12.09	0.15	34	9
Asian#	3.53	0.06	72	0.05	0.83	0	0.52	0.47	0	2
European#	6.35	0.39	5	10.12	0.12	41	9.31	0.16	36	7
Healthy⋇20;	6.69	0.15	40	5.71	0.22	30	4.38	0.36	9	5
Free of CRC⋇20;	1.65	0.44	0	4.35	0.11	54	4.82	0.09	59	3
Free of cancer⋇20;	/	/	/	/	/	/	/	/	/	1
Esophageal	3.20	0.07	69	0.03	0.86	0	0.46	0.50	0	2
Prostate	24.66	0.003	63	22.57	0.007	60	26.18	0.002	66	10
Asian#	1.38	0.50	0	2.80	0.25	29	3.23	0.20	38	3
European#	12.75	0.01	69	16.83	0.002	76	17.47	0.002	77	5
Others#	7.88	0.005	87	0.00	0.95	0	0.88	0.35	0	2
Healthy⋇20;	1.26	0.26	21	0.01	0.94	0	0.22	0.64	0	2
Healthy matched⋇20;	10.02	0.02	70	0.72	0.87	0	2.20	0.53	0	4
Healthy and BPH⋇20;	0.14	0.71	0	0.92	0.34	0	0.43	0.51	0	2
BPH⋇20;	/	/	/	/	/	/	/	/	/	1
BPH and others⋇20;	/	/	/	/	/	/	/	/	/	1
Urothelial	14.28	0.0006	72	9.83	0.04	59	20.37	0.0004	80	5
Asian#	2.30	0.32	13	8.94	0.01	78	16.37	0.0003	88	3
European#	1.22	0.27	18	0.38	0.54	0	0.78	0.38	0	2
Breast	1.68	0.64	0	0.89	0.83	0	0.61	0.89	0	4
Pancreatic	/	/	/	/	/	/	/	/	/	1
Nasopharyngeal	/	/	/	/	/	/	/	/	/	1
Endometrial	/	/	/	/	/	/	/	/	/	1
Cervical	/	/	/	/	/	/	/	/	/	1
Ovarian	/	/	/	/	/	/	/	/	/	1
Lung	/	/	/	/	/	/	/	/	/	1
Oral	/	/	/	/	/	/	/	/	/	1
Liver	/	/	/	/	/	/	/	/	/	1
Thyroid	/	/	/	/	/	/	/	/	/	1
Lymphoma	/	/	/	/	/	/	/	/	/	1
Overall	161.42	⋇20;0.00001	64	138.89	⋇20;0.00001	58	177.76	⋇20;0.00001	67	59

# Stratified by ethnicity, including Asian, European, and others (North American and African).

⋇20;Stratified by controls, including benign prostatic hyperplasia (BPH), BPH or visitors or requesting vasectomy (BPH and others), benign urological patients matched, chronic atrophic gastritis (CAG), free of colorectal cancer (free of CRC), free of cancer, healthy, healthy and BPH, healthy and free of cancer, healthy matched, kindreds, and normal peritumoral tissues.

**Table 3:** Adjusted *R^2^* and corresponding *I^2^* from the meta-regression models.

Covariate	*AA*			*CA*			*(AA+CA)*			No. of datasets
	*I^2^* (%)	Adjusted *R^2^* (%)	*P* value	*I^2^* (%)	Adjusted *R^2^* (%)	*P* value	*I^2^* (%)	Adjusted *R^2^* (%)	*P* value	
Ethnicity#	61	12	0.05	58	−6	0.67	66	−1	0.44	59
Cancer type†	50	46	0.04	46	55	0.08	54	49	0.03	59
Control§	58	25	0.12	46	47	0.01	54	53	0.002	59
Ethnicity and cancer type	46	59	0.02	48	41	0.17	56	41	0.07	59
Ethnicity and control	55	36	0.04	46	41	0.01	53	54	0.001	59
Cancer type and control	50	51	0.15	28	100	0.003	33	100	0.0003	59
Ethnicity, cancer type and control	45	63	0.07	30	80	0.01	33	88	0.001	59

# Ethnicity, including Asian, European, and others (North American and African);

§ Cancer type, including breast, colorectal, esophageal, gastric, gynecological, lung, nasopharyngeal, pancreatic, prostate, urothelial, oral, liver, thyroid and lymphoma;

† Controls, including benign prostatic hyperplasia (BPH), BPH or visitors or requesting vasectomy (BPH and others), benign urological patients matched, chronic atrophic gastritis (CAG), free of colorectal cancer (free of CRC), free of cancer, healthy, healthy and BPH, healthy and free of cancer, healthy matched, and normal peritumoral tissues.

**Figure 1:**
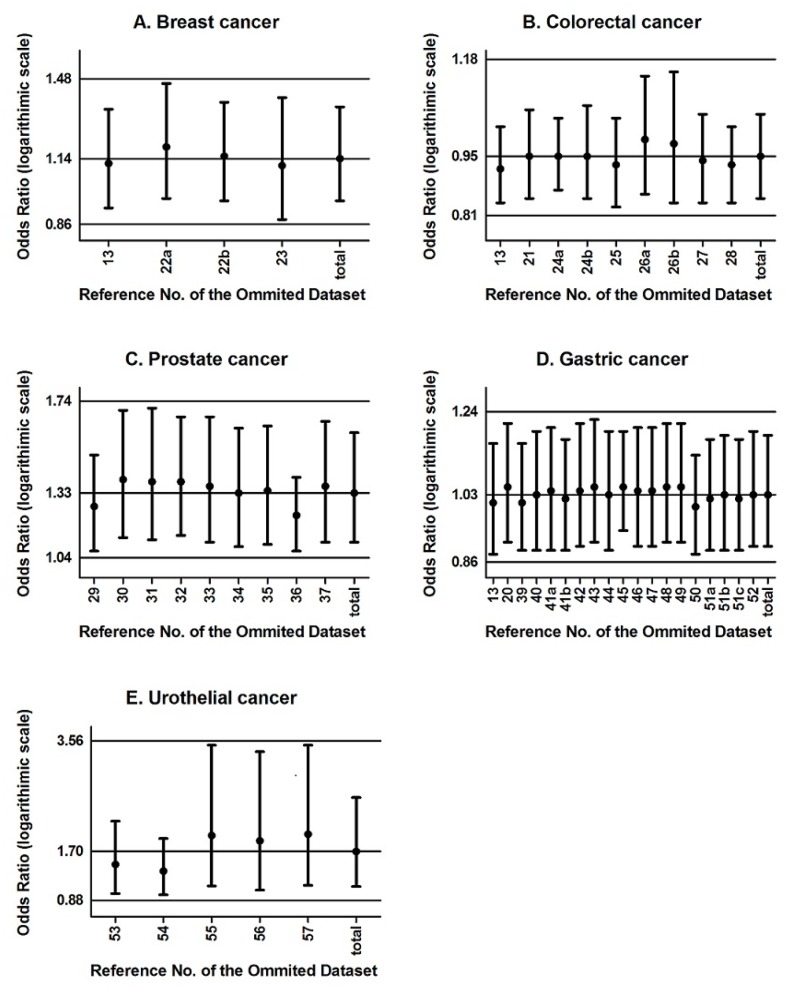
One-way sensitivity analysis for the stability of observations in the meta-analysis. The pooled odds ratios (ORs) and 95% confidence intervals (CIs) of the *-160A* allele carriers are evaluated by comparing to the *CC* genotype, omitting each dataset in each type of cancer (as of March 2012). The pooled ORs are calculated with a random-effects model. The numbers on the *x-*axis refer to the studies extracted. 22a, Sweden; 22b, Czech Republic; 24a, Familial; 24b, Sporadic; 26a, Phase 1; 26b, Phase 2; 41a, Beijing; 41b, Linqu; 51a, Canada; 51b, Germany; 51c, Portugal; total, no dataset omitted.

The Harbord test was used to detect the publication bias of data containing more than three datasets and indicated negligible publication bias (*P*>0.05) in most of the data, except in *-160A* carriers of prostate and urothelial cancers and *CA* heterozygotes of urothelial cancer (Table 4). One-way sensitivity analysis, which was performed by removing one data set at a time, was carried out to confirm the stability of the estimated OR (Figure 1). As shown in Table 5, when the Venice guidelines were applied, cumulative evidence for all cancer types was considered ‘weak.’ Detailed information on the assessment of each cancer type is summarized in Table S3.

**Table 4:** Harbord test of each genotype in different cancer types (as of March 2012) with coefficient and standard error.

Cancer type	*AA*			*CA*			*(AA+CA)*			No. of datasets
	Coef.	Std. err.	*P* value	Coef.	Std. err.	*P* value	Coef.	Std. err.	*P* value	
Breast	0.12	1.60	0.95	−0.15	1.23	0.91	0.08	1.02	0.94	4
Colorectal	−0.17	0.66	0.81	0.94	0.65	0.19	0.80	0.67	0.27	9
Gastric	1.01	0.92	0.29	1.10	0.90	0.24	1.44	0.91	0.13	19
Prostate	2.12	1.09	0.09	3.12	1.42	0.06	3.54	1.43	0.04	10
Urothelial	3.65	5.63	0.56	4.06	0.77	0.01	6.37	1.46	0.02	5
Overall	1.23	0.41	0.004	1.55	0.37	0.000	1.86	0.41	0.000	59

**Table 5:** Assessment of cumulative evidence for the association of the -160C/A polymorphism and different cancers.

Cancer type	*AA*		*CA*		*(AA+CA)*	
	scheme	evidence	scheme	evidence	scheme	evidence
Colorectal	ACB	weak	ACC	weak	ACC	weak
Gastric	BCC	weak	BCC	weak	BCC	weak
Prostate	BCC	weak	BCC	weak	BCC	weak
Urothelial	BCA	weak	BCC	weak	BCC	weak
Breast	BCC	weak	BCC	weak	BCC	weak
Esophageal	CCC	weak	CCB	weak	CCB	weak
Pancreatic	CCB	weak	CCB	weak	CCB	weak
Nasopharyngeal	CCB	weak	CCB	weak	CCB	weak
Endometrial	CCB	weak	CCB	weak	CCB	weak
Cervical	CCB	weak	CCC	weak	CCB	weak
Ovarian	CCB	weak	CCC	weak	CCC	weak
Lung	CCB	weak	CCB	weak	CCB	weak
Oral	CCB	weak	CCB	weak	CCB	weak
Liver	CCB	weak	CCC	weak	CCB	weak
Thyroid	CCB	weak	CCB	weak	CCB	weak
Lymphoma	CCB	weak	CCC	weak	CCC	weak

Compared with our previous study [3], evidence on seven new types of cancer was reported, including pancreatic [11], nasopharyngeal [12], endometrial [13], cervical [13], ovarian [14], oral [15], liver [16], and thyroid [17] cancers and lymphoma [18]. There was no change concerning evidence on lung [19] and esophageal [20], [21] cancers.

### Breast Cancer

One additional study [13] was added to previous breast cancer studies [22], [23], which led to a total of 1,142 cases and 1,063 controls. The *-160A* carriers were still not more susceptible to breast cancer (OR = 1.14, 95% CI = 0.96–1.36) with a fixed-effects model, and no heterogeneity (*Q* = 0.61, *P* = 0.89, *I^2^* = 0%) was detected among these data sets.

### Colorectal Cancer

Six new datasets from four studies [13], [24]–[26] were added to previous data [21], [27], [28], which included 7,117 cases and 7,157 controls altogether. Using a random-effects model, the *-160A* carriers were not more susceptible to colorectal cancer compared with all genotypes (OR = 0.95, 95% CI = 0.85–1.05), and the heterogeneity among seven datasets was moderate (*Q* = 12.09, *P* = 0.15, *I^2^* = 34%). Then, we performed a subgroup analysis stratified by source of controls or ethnicity, and the source of heterogeneity was identified in the colorectal cancer-free control subgroup when it was divided by the source of controls (*Q* = 4.82, *P* = 0.09, *I^2^* = 59%) and in the European subgroup when it was divided by ethnicity (*Q* = 9.31, *P* = 0.16, *I^2^* = 36%). The heterogeneity could be attributed to one dataset from Grunhage et al. [24], in which the association was investigated between the *-160C/A* polymorphism and familial colorectal cancer. After exclusion of this dataset, the heterogeneity was effectively decreased to ‘low’ (*Q* = 8.64, *P* = 0.28, *I^2^* = 19%), and the pooled OR estimated in the fixed-effect model was 0.93 (95% CI = 0.87–0.99, *P* = 0.03). The estimated OR of the *-160AA* homozygote in Europeans was 0.85 (95% CI = 0.74–0.99, P = 0.03), with low heterogeneity (*Q* = 6.35, *P* = 0.39, *I^2^* = 5%), indicating that it played protective roles in colorectal cancer.

### Prostate Cancer

Two additional studies [29], [30] were added to previous prostate cancer studies [31]–[38], resulting in a total of 3,570 cases and 3,304 controls. The genotype distribution in controls from two studies [31], [32] was significantly deviated from Hardy-Weinberg equilibrium (*P*<0.05). After excluding these datasets, the pooled OR estimated in *-160A* carriers was 1.33 (95% CI = 1.18–1.50), indicating the same predisposition to prostate cancer as before excluding these datasets (OR = 1.24, 95% CI = 1.13–1.37). To clarify the possible sources of the significant heterogeneity among these datasets (*Q* = 26.18, *P* = 0.002, *I^2^* = 66%), we performed a subgroup analysis according to the source of controls and ethnicity, respectively. Stratification by source of controls effectively decreased the heterogeneity (*I^2^*_healthy_ = 0%, *I^2^*_healthy-matched_ = 0%, *I^2^*_healthy and benign prostatic hyperplasia_ = 0%); however, this decrease may also be due to a reduction in power for the Q-test. When stratified by ethnicity, the *-160A* allele was revealed to be an ethnicity-dependent risk factor for prostate cancer. ORs estimated using the random-effects model were greater than 1.0 for both Asians (OR = 1.56, 95% CI = 1.16–2.08) and Europeans (OR = 1.25, 95% CI = 1.02–1.55), while no relationship was found between the *-160A* allele and the progression of prostate cancer in North Americans (OR = 1.10, 95% CI = 0.86–1.41).

### Gastric Cancer

Eight datasets from seven studies [13], [39]–[44] were added to our previous report [20], [45]–[52], and there were 3,453 patients and 4,775 controls altogether. The exclusion of two studies [20], [45], in which genotype distribution significantly deviated from Hardy-Weinberg equilibrium, revealed no predisposition of the *-160A* allele to gastric cancer (OR = 1.05, 95% CI = 0.95–1.16). The heterogeneity among these datasets was moderate (*Q* = 33.44, *P* = 0.01, *I^2^* = 46%), and stratification by ethnicity explained the source of heterogeneity. This stratification showed that heterogeneity in the European subgroup was significant (*Q* = 13.29, *P* = 0.02, *I^2^* = 62%). This finding might be mainly attributed to the dataset from Humar et al. [50], in which diffuse gastric cancer was investigated. As a special histological form of gastric cancer, diffuse gastric cancer was more prevalent in younger age groups. The heterogeneity was effectively removed after exclusion of this dataset (*Q* = 6.49, *P* = 0.17, *I^2^* = 38%), as expected. The OR estimated for the *-160A* carriers was 1.03 (95% CI = 0.90–1.18) in pooled datasets, 0.93 (95% CI = 0.80–1.08) in Asians, 1.20 (95% CI = 0.91–1.58) in Europeans, and 1.53 (95% CI = 0.99–2.36) in others. The association of *-160A* allele carriers with the progression of gastric cancer in Europeans disappeared, and the relationship between the *-160A* allele and gastric cancer was not detectable.

### Urothelial Cancer

Five studies [53]–[57], including two updated reports [54], [57], detected the *-160C/A* polymorphism in urothelial cancer patients, which involved 1,064 cases and 1,124 controls. Overall, the meta-analysis showed that the *-160A* carriers had a significantly increased risk of developing urothelial cancer (OR = 1.70, 95% CI = 1.11–2.61), and significant heterogeneity was found among the five studies (*Q* = 20.37, *P* = 0.0004, *I^2^* = 80%). Indeed, the genotype distribution of two studies [53], [54] significantly deviated from Hardy-Weinberg equilibrium. Exclusion of these data sets successfully reduced the heterogeneity (*Q* = 0.87, *P* = 0.65, *I^2^* = 0%), and the pooled OR calculated in the fixed-effects model no longer indicated predisposition in *-160A* carriers (OR = 1.18, 95% CI = 0.98–1.43). However, ORs estimated in *AA* homozygotes (OR = 1.64, 95% CI = 1.05–2.56) still revealed a higher risk for the development of urothelial cancer, and heterogeneity between studies was low (*Q* = 2.55, *P* = 0.28, *I^2^* = 22%).

### Other Cancers

Single studies investigated the association between *-160A* carriers and lung [19] (OR = 2.81, 95% CI = 1.36–5.83, *P* = 0.0005), nasopharyngeal [12] (OR = 2.02, 95% CI = 1.20–3.41, *P* = 0.0008), thyroid [17] (OR = 2.35, 95% CI = 1.39–3.99, *P* = 0.0001), endometrial [13] (OR = 1.93, 95% CI = 1.19–3.14, *P* = 0.0008), oral [15] (OR = 0.57, 95% CI = 0.41–0.80, *P* = 0.0001), pancreatic [11] (OR = 1.62, 95% CI = 2.63, *P* = 0.05), liver [16] (OR = 0.85, 95% CI = 0.56–1.29, *P* = 0.44), cervical [13] (OR = 1.22, 95% CI = 0.77–1.95, *P* = 0.39), and ovarian [14] (OR = 0.93, 95% CI = 0.63–1.37, *P* = 0.71) cancer and lymphoma [18] (OR = 0.91, 95% CI = 0.51–1.60, *P* = 0.74). The exact number of cases and controls across datasets for each cancer type is shown in Table 1. The estimated OR indicated that the *-160A* allele of the *E-cadherin* gene provided a higher risk for the development of lung, nasopharyngeal, thyroid, endometrial and oral cancer, but the credibility of these associations was considered ‘weak’ after application of the Venice interim guidelines [9]. Because of the significance of the *-160C/A* polymorphism in human cancers, much more data will be provided in the future to enhance the statistical power in these cancer types.

### Discussion

The meta-analysis performed in this paper indicated that the *-160AA* homozygote predisposed its carriers to urothelial cancer. Carriers of the *-160A* allele had an increased risk of prostate cancer. The ethnicity-dependent susceptibility of *-160A* carriers to gastric cancer [3] disappeared with the inclusion of updated evidence, whereas susceptibility was demonstrated in prostate cancer. The credibility of single studies that investigated the association of the *-160A* allele with lung, nasopharyngeal, pancreatic, thyroid, endometrial and oral cancer was considered ‘weak,’ which requires further verification. No evidence was found that the *-160A* allele predisposed its carriers to breast, colorectal, esophageal, gynecological, gastric, or liver cancer or lymphoma.

The meta-analysis, which is not maintained, may become out of date or misleading. Bias and greater heterogeneity arose because of the further inclusion of new evidence, which suggests the requirement for more studies concerning the *-160C/A* polymorphism and cancer risk, especially those with rigorous selection of case and control samples and the reporting of more studies with a large sample size and negative results. In addition to publication bias, which is popular in meta-analyses, different mechanisms can lead to asymmetry in funnel plots, including true heterogeneity resulting from improper study design [58].

The authors combined case-control studies, which are relatively more practical and inexpensive than prospective cohort studies in the investigation of relationships between suspected risk factors and diseases, especially those with low incidence, such as cancers. However, the crucial concern in the design of case-control studies is choosing case and control samples, especially a proper control population, given the explicit diagnostic criteria for cancers. Ideal controls should be a general group of persons without the disease of interest, from which qualified cases arise once diagnosed. This general group does not exclude those with other kinds of disease, whereas no relationship should be expected between the healthy status of the control and the investigated ‘risk factor’ because the correlation may exaggerate or underestimate the overall estimated OR [59].

Controls selected in studies investigating the association between the *-160C/A* polymorphism and prostate cancer risk could be divided into healthy [30], [32], healthy matched [31], [33], [35], [38], benign prostatic hyperplasia [29], healthy and benign prostatic hyperplasia [34], [37] and benign prostatic hyperplasia or others [36]. Subsequent subgroup analysis stratified by controls in data sets of prostate cancer indicated homogeneity in each strata, indicating that the between-study variance in the prostate subgroup resulted from different controls. However, it should also be noted that the reduced heterogeneity may also result from a reduction in power for the Q-test because of the small sample size in some subgroups.

Furthermore, a question arose because of the low expression level of E-cadherin in benign prostatic hyperplasia [60], [61] and urothelial diseases [62], [63], which could also have resulted from the *-160A* polymorphism in the promoter region of *E-cadherin*. If the relationship between the *-160C/A* polymorphism of *E-cadherin* and benign prostatic hyperplasia and other urothelial diseases could not be excluded, the selection of patients with these diseases as controls may not be suitable. We tested Hardy-Weinberg equilibrium at the polymorphism site in the control samples, and deviation could be a symptom of disease association [64]. However, there was no guarantee that following Hardy-Weinberg equilibrium excluded a relationship between allele distribution and susceptible diseases [65].

Deviation from Hardy-Weinberg equilibrium in a random sample could be due to inbreeding, population stratification, or selection, and may be indicative of problematic assays [64], [65]. Heterogeneity in evidence concerning urothelial cancer was successfully reduced to zero after the exclusion of studies that significantly deviated from Hardy-Weinberg equilibrium, what may indicate an inappropriate choice of control samples in those studies. We observed that the estimated OR qualitatively changed with or without excluding studies in *-160A* carriers, although it was maintained in *AA* homozygotes. It may not be necessary to consider deviation from Hardy-Weinberg equilibrium in patients, while it should be a prerequisite for control or random samples to be in Hardy-Weinberg equilibrium.

The Harbord test detected significant publication bias in *-160A* carriers with urothelial cancer. However, excluding two data sets [53], [54] that deviated from Hardy-Weinberg equilibrium effectively removed not only the significant heterogeneity but also the publication bias (*P* = 0.166). Additionally, the estimated pooled OR no longer indicated any predisposition in *-160A* carriers (OR = 1.18, 95% CI = 0.98–1.43). Significant publication bias was also detected in *-160A* carriers with prostate cancer by the Harbord test, indicating ‘small-study effects’ in the data. Small studies are systematically biased toward a higher association, which may due to either their poor methodological quality or their biased choice of high-risk groups [8]. More studies are needed to properly address the bias of the existing data concerning *-160A* carriers and prostate cancer, especially studies with a large sample size and negative results.

We noted that excluding the dataset of Grunhage et al. [24] from data concerning colorectal cancer reduced its moderate heterogeneity to low, and the overall estimated ORs were quantitatively changed. The excluded dataset investigated the association between the *-160C/A* polymorphism and familial colorectal cancer, which is a specific type of cancer accounting for approximately 20% of colorectal cancer. The strong family history suggested additional inherited susceptibility factors that are yet to be defined [24]. The inherent difference between familial and sporadic colorectal cancer may lead to stratification of case samples; however, further subgroup analysis stratified by these types was not applicable due to unavailability of the exact type of each case from the original data.

In summary, the combined analysis of these case-control studies indicated that *-160A* of the *E-cadherin* gene is a tumor susceptibility allele for the development of urothelial and prostate cancers; however, this conclusion is based on unadjusted results, and more studies are needed. The *AA* homozygote carriers are at a higher risk for the development of prostate and urothelial cancers. The association between the *-160A* allele and lung, nasopharyngeal, thyroid, endometrial and oral cancer indicated by single studies needs further validation.

## Supporting Information

Figure S1 **The flow diagram for the review process and outcomes of inclusion and exclusion.**
(TIF)

Figure S2 **Meta-analysis of** ***-160A*** **association with fourteen types of cancers (as of March 2012).** Odds ratios (ORs) and 95% confidence intervals (CIs) are displayed at a logarithmic scale. Events and total represent the number of *-160A* allele carriers and all the genotypes respectively.
(TIF)

Table S1 **Characteristics of the 47 case-control studies included in this meta-analysis.**
(DOC)

Table S2 **Distribution of three genotypes at the** ***E-cadherin -160C/A*** **polymorphic site among case and control samples from 47 case-control studies in this meta-analysis.**
(DOC)

Table S3 **Detailed information on the assessment of evidence in each cancer type.**
(DOC)
