# Supplementary figures and images for: Contribution of the -160C/A Polymorphism in the E-cadherin Promoter to Cancer Risk: A Meta-Analysis of 47 Case-Control Studies
Source: PLoS One. 2012 Jul 5;7(7):e40219. doi: 10.1371/journal.pone.0040219 (PMC3390351; doi:10.1371/journal.pone.0040219)

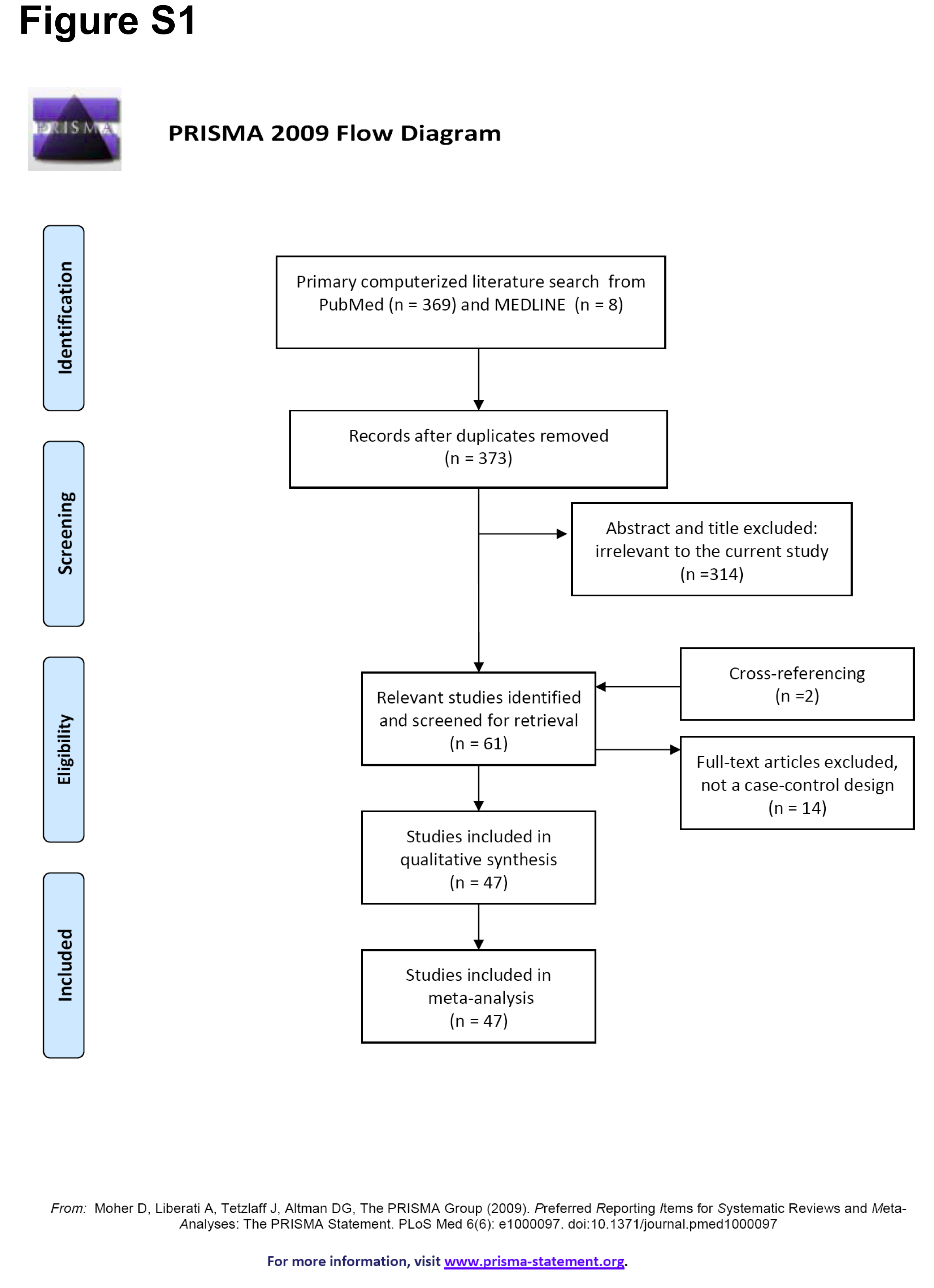

Supplement: Figure S1 — The flow diagram for the review process and outcomes of inclusion and exclusion. (TIF) [file pone.0040219.s001.tif]

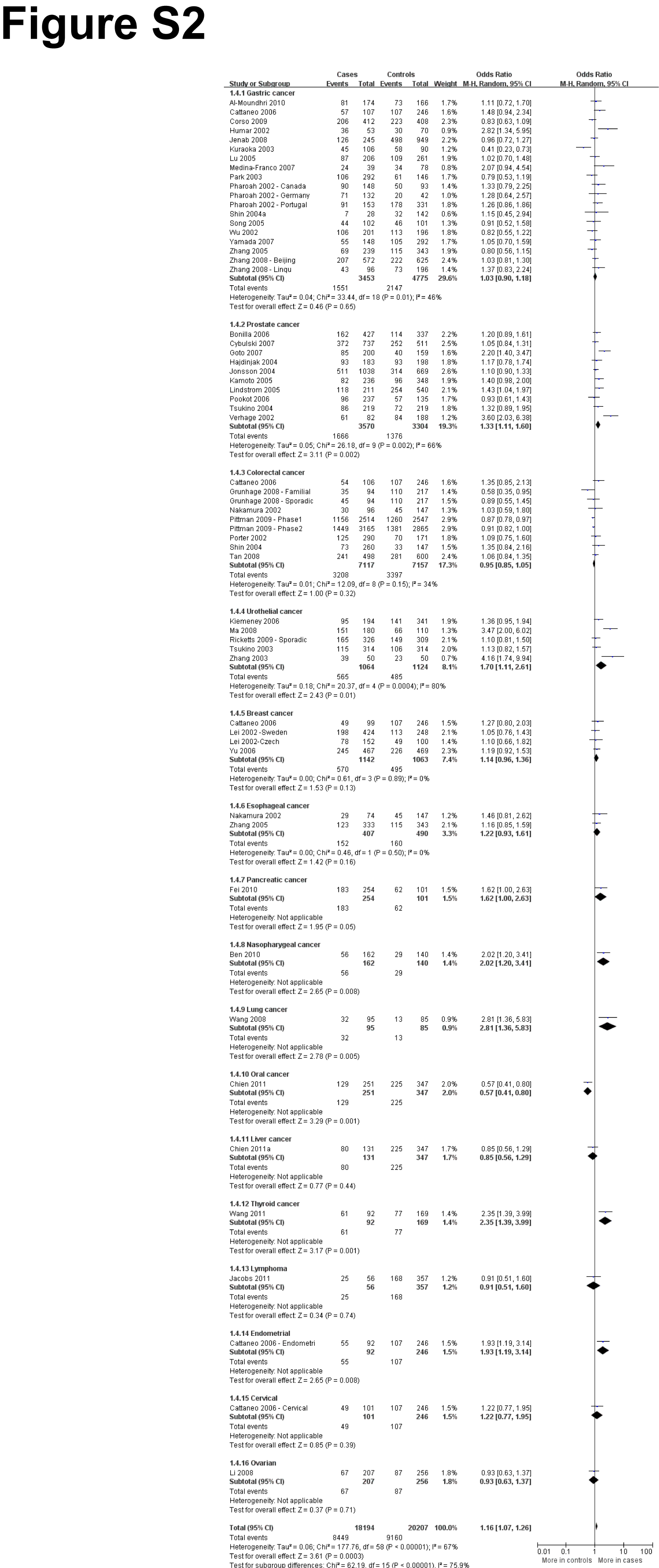

Supplement: Figure S2 — Meta-analysis of -160A association with fourteen types of cancers (as of March 2012). Odds ratios (ORs) and 95% confidence intervals (CIs) are displayed at a logarithmic scale. Events and total represent the number of -160A allele carriers and all the genotypes respectively. (TIF) [file pone.0040219.s002.tif]
