# Supplementary material for: Contribution of the -160C/A Polymorphism in the E-cadherin Promoter to Cancer Risk: A Meta-Analysis of 47 Case-Control Studies
Source: PLoS One. 2012 Jul 5;7(7):e40219. doi: 10.1371/journal.pone.0040219 (PMC3390351; doi:10.1371/journal.pone.0040219)
Supplement: Table S1 — Characteristics of the 47 case-control studies included in this meta-analysis. (DOC) [file pone.0040219.s003.doc]

**Table S1** Characteristics of the 47 case-control studies included in this meta-analysis

| First author and year (ref. no.) | Country of origin | Racial descent | Cancer types | No. of cases /  no. of controls | Characteristics of controls | Genotyping |
| --- | --- | --- | --- | --- | --- | --- |
| Lei 2002^22^ | Sweden | European | Breast | 424/248 | Mothers of patients not expected to have a statistically different prevalence of breast cancer than the general population | PCR-SSCP |
|  | Czech | European | Breast | 152/100 | Healthy | PCR-SSCP |
| Yu 2006^23^ | China | Asian | Breast | 468/470 | Healthy | TaqMan-assay |
| Nakamura 2002^21^ | Japan | Asian | Esophageal | 74/147 | Healthy | PCR-SSCP |
|  |  |  | Colorectal | 96/147 | Healthy | PCR-SSCP |
| Zhang 2005^20^ | China | Asian | Esophageal | 333/343 | Healthy, hospital-based | PCR-RFLP |
|  |  |  | Gastric | 239/343 |  | PCR-RFLP |
| Shin 2004^52^ | Korea | Asian | Gastric | 28/142 | Healthy | PCR-RFLP |
| Wu 2002^49^ | China | Asian | Gastric | 201/196 | Healthy, matched for age, gender and ethnicity | PCR-RFLP |
| Humar 2002^50^ | Italy | European | Gastric | 53/70 | Healthy, matched for age, gender and residence | PCR-RFLP |
| Pharoah 2002^51^ | Canada | European | Gastric | 148/93 | Healthy | PCR-RFLP |
|  | Germany | European | Gastric | 132/42 | Healthy | PCR-RFLP |
|  | Portugal | European | Gastric | 153/331 | Healthy | PCR-SSCP |
| Kuraoka 2003^45^ | Japan | Asian | Gastric | 106/90 | Healthy | PCR-SSCP |
| Park 2003^48^ | Korea | Asian | Gastric | 292/146 | Healthy | PCR-SSCP |
| Song 2005^46^ | China | Asian | Gastric | 102/101 | Healthy, matched for age and gender | PCR-DHPLC |
| Lu 2005^47^ | China | Asian | Gastric | 206/261 | Healthy | PCR-RFLP |
| Jenab 2008^42^ | France | European | Gastric | 245/949 | Free of cancer | TaqMan-assay |
| Al-Moundhri 2010^42^ | Oman | Omani | Gastric | 174/166 | Healthy | PCR with an ABI premix |
| Zhang 2008^41^ | China | Chinese | Gastric (Linqu) | 96/196 | Chronic astrophic gastritis | PCR-RFLP |
|  |  |  | Gastric (Beijing) | 572/625 | Free of cancer |  |
| Medina-Franco 2007^39^ | Mexico | Mexican | Gastric | 39/78 | Healthy | PCR-SSCP |
| Corso 2009^43^ | Italy | Italian | Gastric | 412/408 | Healthy | PCR-RFLP |
| Yamada 2007^40^ | Japan | Japanese | Gastric | 148/292 | Free of cancer | PCR-RFLP |
| Cattaneo 2006^13^ | Italy | Italian | Gastric | 107/246 | Free of cancer | PCR |
|  |  |  | Breast | 99/246 |  |  |
|  |  |  | Cervical | 101/246 |  |  |
|  |  |  | Endometrial | 92/246 |  |  |
|  |  |  | Colorectal | 106/246 |  |  |
| Pittman 2009^26^ | UK | British Caucasian | Colorectal  (Phase1) | 3，670/3，807 | Healthy | AS-PCR |
|  |  |  | Colorectal  (Phase2) | 4，614/4，246 |  |  |
| Grünhage 2008^24^ | Germany | European | Colorectal  (Familial) | 94/217 | Free of colorectal cancer | PCR-RFLP |
|  |  |  | Colorectal  (Sporatic) | 94/217 |  |  |
| Tan 2008^25^ | German | Germany | Colorectal | 498/600 | Free of colorectal cancer | PCR-RFLP |
| Porter 2002^27^ | UK | European | Colorectal | 290/171 | Healthy | PCR-RFLP |
| Shin 2004^28^ | Korea | Asian | Colorectal | 260/147 | Healthy, hospital-based | PCR-RFLP |
| Kamoto 2005^34^ | Japan | Asian | Prostate | 236/348 | Hospital-based, with negative PSA and DRE^†^ | PCR-RFLP |
| Verhage 2002^36^ | Netherlands | European | Prostate | 82/188 | Benign prostatic hyperplasia, or visitors, or requesting vasectomy | PCR-RFLP |
| Tsukino 2004^35^ | Japan | Asian | Prostate | 219/219 | Healthy, matched for age | PCR-RFLP |
| Hajdinjak 2004^37^ | Slovenia | European | Prostate | 183/198 | Healthy (n=168), Benign prostatic hyperplasia (n=30) | TaqMan-assay |
| Jonsson 2004^31^ | Sweden | European | Prostate | 1,038/669 | Healthy, matched for age, gender and residence | TaqMan-assay |
| Lindström 2005^38^ | Sweden | European | Prostate | 211/540 | Healthy, matched for age, gender and residence | DASH |
| Cybulski 2007^30^ | Poland | Polish | Prostate | 737/511 | Healthy elderly men aged 50 and above | RFLP-PCR |
| Pookot 2006^32^ | America | European | Prostate | 237/135 | Healthy | PCR-RFLP |
| Bonilla 2006^33^ | America | European | Prostate | 427/337 | Healthy, matched for age and ethnicity | PCR-RFLP |
| Goto 2007^29^ | Japan | Japanese | Prostate | 200/159 | Benign prostatic hyperplasia | PCR-RFLP |
| Tsukino 2003^55^ | Japan | Asian | Urothelial | 314/314 | Healthy, matched for age and gender | PCR-RFLP |
| Zhang 2003^53^ | China | Asian | Urothelial | 50/50 | Benign urological patients, matched for age and gender | PCR-RFLP |
| Kiemeney 2006^56^ | Netherlands | European | Urothelial | 194/341 | Healthy | PCR-RFLP |
| Ricketts 2009^57^ | UK | European | Urothelial | 326/309 | Healthy-matched | AS-PCR |
| Ma 2008^54^ | China | Chinese | Urothelial | 180/110 | Healthy and free of cancer | PCR |
| Wang 2008^19^ | China | Asian | Lung | 95/85 | Healthy | PCR-RFLP |
| Ben 2010^12^ | Tunisia | Tunisian | Nasopharyngeal | 162/140 | Healthy | PCR-RFLP |
| Fei 2010^11^ | China | Chinese | Pancreatic | 254/101 | Healthy | PCR-RFLP  DHPLC |
| Li 2008^14^ | China | Chinese | Ovarian | 207/256 | Free of cancer | PCR-RFLP |
| Chien 2012^15^ | Taiwan | Chinese | Oral | 251/347 | Free of cancer | RFLP |
| Chien 2011^16^ | Taiwan | Chinese | Liver | 131/347 | Healthy-matched | RFLP |
| Wang 2011^17^ | China | Chinese | Thyroid | 92/169 | Healthy | PCR |
| Jacobs 2011^18^ | Germany | European | Lymphoma | 56/361 | Healthy-matched | TaqMan-assay |
