# Supplementary material for: Contribution of the -160C/A Polymorphism in the E-cadherin Promoter to Cancer Risk: A Meta-Analysis of 47 Case-Control Studies
Source: PLoS One. 2012 Jul 5;7(7):e40219. doi: 10.1371/journal.pone.0040219 (PMC3390351; doi:10.1371/journal.pone.0040219)
Supplement: Table S2 — Distribution of three genotypes at the E-cadherin -160C/A polymorphic site among case and control samples from 47 case-control studies in this meta-analysis. (DOC) [file pone.0040219.s004.doc]

**Table S2** Distribution of three genotypes at the *E-cadherin* *-160C/A* polymorphic site among case and control samples from 47 case-control studies in this meta-analysis

| First author and year (ref. no.) | Cancer types | AA | |  | CA | |  | CC | | |  | |
| --- | --- | --- | --- | --- | --- | --- | --- | --- | --- | --- | --- | --- |
|  |  | Case (%) | Control (%) |  | Case (%) | Control (%) |  | Case (%) | Control (%) | |  | |
| Lei^22^ | Breast * | 32 (7.5) | 21 (8.5) |  | 166 (39.2) | 92 (37.1) |  | 226 (53.3) | 135 (54.4) | | |  |
|  | Breast ^†^ | 18 (11.8) | 7 (7.0) |  | 60 (39.5) | 42 (42.0) |  | 74 (48.7) | 51 (51.0) | |  | |
| Yu^23^ | Breast | 44 (9.4) | 39 (8.3) |  | 201 (43.0) | 187 (39.9) |  | 222 (47.5) | 243 (51.8) |  | | |
| Nakamura^21^ | Colorectal | 0 (0) | 5 (3.4) |  | 30 (31.2) | 40 (27.2) |  | 66 (68.8) | 102 (69.2) |  | | |
| Porter^27^ | Colorectal | 10 (3.4) | 15 (8.8) |  | 115 (39.7) | 55 (32.2) |  | 165 (56.9) | 101 (59.1) |  | | |
| Shin^28^ | Colorectal | 7 (2.7) | 1 (0.7) |  | 66 (25.4) | 32 (21.8) |  | 187 (71.9) | 114 (77.5) |  | | |
| Nakamura^21^ | Esophageal | 5 (6.8) | 5 (3.4) |  | 24 (32.4) | 40 (27.2) |  | 45 (60.8) | 102 (69.2) |  | | |
| Zhang^20^ | Esophageal | 10 (3.0) | 19 (5.5) |  | 113 (33.9) | 96 (28.0) |  | 210 (63.1) | 228 (66.5) |  | | |
| Zhang^20^ | Gastric | 7 (2.9) | 19 (5.5) |  | 62 (26.0) | 96 (28.0) |  | 170 (71.1) | 228 (66.5) | | | |
| Shin^52^ | Gastric | 1 (3.6) | 1 (0.7) |  | 6 (21.4) | 31 (21.8) |  | 21 (75.0) | 110 (77.5) | | | |
| Wu^49^ | Gastric | 4 (2.0) | 19 (9.7) |  | 102 (50.7) | 94 (48.0) |  | 95 (47.3) | 83 (42.3) | | | |
| Humar^50^ | Gastric | 10 (18.9) | 3 (4.3) |  | 26 (49.1) | 27 (38.6) |  | 17 (32.1) | 40 (57.1) | | | |
| Pharoah^51^ | Gastric ^‡^ | 14 (9.5) | 6 (6.5) |  | 76 (51.4) | 44 (47.3) |  | 58 (39.2) | 43 (46.2) | | | |
|  | Gastric ^§^ | 13 (9.8) | 5 (11.9) |  | 58 (43.9) | 15 (35.7) |  | 61 (46.2) | 22 (52.4) | | | |
|  | Gastric ^#^ | 11 (7.2) | 27 (8.2) |  | 80 (52.3) | 151 (45.6) |  | 62 (40.5) | 153 (46.2) | | | |
| Kuraoka^45^ | Gastric | 11 (10.4) | 6 (6.7) |  | 34 (32.1) | 52 (57.8) |  | 61 (57.5) | 32 (35.6) | | | |
| Park^38^ | Gastric | 14 (4.8) | 6 (4.1) |  | 92 (31.5) | 55 (37.7) |  | 186 (63.7) | 85 (58.2) | | | |
| Song^46^ | Gastric | 6 (5.9) | 5 (4.9) |  | 38 (37.2) | 41 (40.6) |  | 58 (56.9) | 55 (54.5) | | | |
| Lu^47^ | Gastric | 12 (5.8) | 18 (6.9) |  | 75 (36.4) | 91 (34.9) |  | 119 (57.8) | 152 (58.2) | | | |
| Kamoto^34^ | Prostate | 11 (4.7) | 11 (3.2) |  | 71 (30.0) | 85 (24.4) |  | 154 (65.3) | 252 (72.4) | | | |
| Verhage^36^ | Prostate | 3 (3.7) | 9 (4.8) |  | 58 (70.7) | 75 (39.9) |  | 21 (25.6) | 104 (55.3) | | | |
| Tsukino^35^ | Prostate | 9 (4.1) | 6 (2.7) |  | 77 (35.2) | 66 (30.1) |  | 133 (60.7) | 147 (67.1) | | | |
| Hajdinjak^37^ | Prostate | 21 (11.5) | 12 (6.1) |  | 72 (39.3) | 81 (40.9) |  | 90 (49.2) | 105 (53.0) | | | |
| Jonsson^31^ | Prostate | 90 (8.7) | 65 (9.7) |  | 421 (40.6) | 249 (37.2) |  | 527 (50.8) | 355 (53.1) | | | |
| Lindström^38^ | Prostate | 25 (11.8) | 33 (6.1) |  | 93 (44.1) | 221 (40.9) |  | 93 (44.1) | 286 (53.0) | | | |
| Pookot^32^ | Prostate | 16 (6.7) | 18 (13.3) |  | 80 (33.8) | 39 (28.9) |  | 141 (59.5) | 78 (57.8) | | | |
| Bonilla^33^ | Prostate | 19 (4.4) | 6 (1.8) |  | 143 (33.5) | 108 (32.0) |  | 265 (62.1) | 223 (66.2) | | | |
| Tsukino^55^ | Urothelial | 21 (6.7) | 9 (2.9) |  | 94 (29.9) | 97 (30.9) |  | 199 (63.4) | 208 (66.2) | | | |
| Zhang^53^ | Urothelial | 22 (44.0) | 11 (22.0) |  | 17 (34.0) | 12 (24.0) |  | 11 (22.0) | 27 (54.0) | | | |
| Kiemeney^56^ | Urothelial | 18 (9.3) | 19 (5.6) |  | 77 (39.7) | 122 (35.8) |  | 99 (51.0) | 200 (58.6) | | | |
| Wang^19^ | Lung | 5 (5.3) | 0 (0) |  | 27 (28.4) | 13 (15.3) |  | 63 (66.3) | 72 (84.7) | | | |
| Medina-Franc^39^ | Gastric | 8 (20.5) | 4 (5.1) |  | 16 (41.0) | 30 (38.5) |  | 15 (38.5) | 44 (56.4) | | | |
| Ma^54^ | Bladder | 90 (50) | 26 (23.6) |  | 61 (33.9) | 40 (36.4) |  | 29 (16.1) | 44 (40.0) | | | |
| Zhang^41^ | Gastric(Linqu) | 5 (5.2) | 8 (4.1) |  | 38 (39.6) | 65 (33.2) |  | 53 (55.2) | 123 (62.8) | | | |
|  | Gastric(Beijing) | 34 (5.9) | 28 (4.5) |  | 173 (30.2) | 194 (31.0) |  | 365 (63.8) | 403 (64.5) | | | |
| Fei^11^ | Pancreatic | 55 (21.7) | 12 (11.9) |  | 128 (50.4) | 50 (49.5) |  | 71 (28) | 39 (38.6) | | | |
| Ben^12^ | Nasopharyngeal | 11 (6.8) | 3 (2.1) |  | 45 (27.8) | 26 (18.6) |  | 106 (65.4) | 111 (79.3) | | | |
| Jenab^42^ | Gastric | 25 (10.2) | 90 (9.5) |  | 101 (41.2) | 408 (43.0) |  | 119 (48.6) | 451 (47.5) | | | |
| Corso^43^ | Gastric | 43 (10.4) | 38 (9.4) |  | 163 (39.6) | 185 (45.3) |  | 206 (50.0) | 185 (45.3) | | | |
| Li^14^ | Ovarian | 4 (2.0) | 7 (2.7) |  | 63 (30.4) | 80 (31.3) |  | 140 (67.6) | 169 (66.0) | | | |
| Pittman^26^ | Colorectal  (phase 1) | 175 (7.0) | 191 (7.5) |  | 981 (39.0) | 1，069 (42.0) |  | 1，358 (54.0) | 1，287 (50.5) | | | |
|  | Colorectal  (phase 2) | 225 (7.1) | 229 (8.0) |  | 1，224 (38.7) | 1152 (40.2) |  | 1，716 (54.2) | 1，484 (51.8) | | | |
| Tan^25^ | Colorectal | 32 (6.27) | 38 (6.33) |  | 209 (41.58) | 243 (40.50) |  | 257 (52.15) | 319 (53.17) | | | |
| Grünhage^24^ | Colorectal (familial) | 8 (8.5) | 24 (22.4) |  | 27(28.7) | 86 (39.6) |  | 59 (62.8) | 107 (49.3) | | | |
|  | Colorectal (sporadic) | 7 (7.4) | 24 (22.4) |  | 38 (40.4) | 86 (39.6) |  | 49 (52.1) | 107 (49.3) | | | |
| Ricketts^57^ | Renal | 29 (8.9) | 25 (8.1) |  | 136 (41.7) | 124 (40.1) |  | 161 (49.4) | 160 (51.8) | | | |
| Yamada^40^ | Gastric | 4 (2.7) | 12 (4.11) |  | 51 (34.46) | 93 (31.85) |  | 93 (62.84) | 187 (64.04) | | | |
| Goto^29^ | Prostate | 6 (3.0) | 1 (0.63) |  | 79 (39) | 39 (25) |  | 115 (58) | 119 (75) | | | |
| Al-Moundhri^42^ | Gastric | 21 (12.0) | 8 (4.8) |  | 60 (34.5) | 65 (39.2) |  | 93 (53.6) | 93 (56.0) | | | |
| Cattaneo^13^ | Colorectal | 10 (9.4) | 18 (7.3) |  | 44 (41.5) | 89 (36.2) |  | 52 (49.1) | 139 (56.5) | | | |
|  | Gastric | 6 (5.6) | 18 (7.3) |  | 51 (47.7) | 89 (36.2) |  | 50 (46.7) | 139 (56.5) | | | |
|  | Cervical | 14 (13.9) | 18 (7.3) |  | 35 (34.7) | 89 (36.2) |  | 52 (51.5) | 139 (56.5) | | | |
|  | Endometrial | 6 (6.5) | 18 (7.3) |  | 49 (53.3) | 89 (36.2) |  | 37 (40.2) | 139 (56.5) | | | |
|  | Breast | 6 (6.1) | 18 (7.3) |  | 43 (43.4) | 89 (36.2) |  | 50 (50.5) | 139 (56.5) | | | |
| Cybulski^30^ | Prostate | 56 (7.6) | 50 (9.8) |  | 316 (42.9) | 202 (39.5) |  | 365 (49.5) | 259 (50.7) | | | |
| Chien^15^ | Oral | 19 (7.6) | 59 (17.0) |  | 110 (43.8) | 166 (47.8) |  | 122 (48.6) | 122 (35.2) | | | |
| Chien^16^ | Liver | 19 (14.5) | 59 (17.0) |  | 61 (46.6) | 166 (47.8) |  | 51 (38.9) | 122 (35.2) | | | |
| Wang^17^ | Thyroid | 12 (13.0) | 17 (10.1) |  | 49 (53.3) | 60 (35.5) |  | 31 (33.7) | 92 (54.4) | | | |
| Jacobs^18^ | Lymphoma | 3 (5.4) | 26 (7.3) |  | 22 (39.3) | 142 (39.8) |  | 31 (55.4) | 189 (52.9) | | | |
| Total | Mixed | 1，422 (7.8) | 1，495 (7.4) |  | 7，027 (38.6) | 7，665 (37.9) |  | 9，745 (53.6) | 11，047 (54.7) | | | |
