# Supplementary material for: Contribution of the -160C/A Polymorphism in the E-cadherin Promoter to Cancer Risk: A Meta-Analysis of 47 Case-Control Studies
Source: PLoS One. 2012 Jul 5;7(7):e40219. doi: 10.1371/journal.pone.0040219 (PMC3390351; doi:10.1371/journal.pone.0040219)
Supplement: Table S3 — Detailed information on the assessment of evidence in each cancer type. (DOC) [file pone.0040219.s005.doc]

**Table S3** Detailed information on the assessment of evidence in each cancer type

|  | Amount  (n_minor_, f_minor_) | Replication | Protection  from Bias | Cumulative evidence |
| --- | --- | --- | --- | --- |
| Colorectal | | | | |
| *AA* | A (1，019, 0.275) | C (no association) | B (nominal OR: 0.85, *P*=0.81) | ACB |
| *CA* | A (1，019, 0.275) | C (no association) | C (nominal OR: 0.97) | ACC |
| *(AA+CA)* | A (1，019, 0.275) | C (no association) | C (nominal OR: 0.95) | ACC |
| Gastric | | | | |
| *AA* | B (570, 0.258) | C (no association) | C (nominal OR: 1.14) | BCC |
| *CA* | B (570, 0.258) | C (no association) | C (nominal OR: 1.01) | BCC |
| *(AA+CA)* | B (570, 0.258) | C (no association) | C (nominal OR: 1.03) | BCC |
| Prostate | | | | |
| *AA* | B (620, 0.234) | C (no association) | C (nominal OR: 1.36, Harbord *P*=0.09) | BCC |
| *CA* | B (620, 0.234) | C (*I^2^* 60) | C (nominal OR: 1.32, Harbord *P*=0.06) | BCC |
| *(AA+CA)* | B (620, 0.234) | C (*I^2^* 66) | C (nominal OR: 0.85, Harbord *P*=0.04) | BCC |
| Urothelial | | | | |
| *AA* | B (270, 0.256) | C (*I^2^* 72) | A (nominal OR:2.58) | BCA |
| *CA* | B (270, 0.256) | C (no association) | C (nominal OR:1.54, Harbord *P*=0.01) | BCC |
| *(AA+CA)* | B (270, 0.256) | C (*I^2^* 80) | C (nominal OR: 1.70, Harbord *P*=0.02; stastical significance was lost with excluding of studies violated from HWE) | BCC |
| Breast | | | | |
| *AA* | B (185, 0.273) | C (no association) | C (nominal OR: 1.14) | BCC |
| *CA* | B (185, 0.273) | C (no association) | C (nominal OR: 1.14) | BCC |
| *(AA+CA)* | B (185, 0.273) | C (no association) | C (nominal OR: 1.14) | BCC |
| Esophageal | | | | |
| *AA* | C (39, 0.188) | C (no association) | C (nominal OR: 1.03) | CCC |
| *CA* | C (39, 0.188) | C (no association) | B (no obvious bias but no bias diagnostics) | CCB |
| *(AA+CA)* | C (39, 0.188) | C (no association) | B (no obvious bias but no bias diagnostics) | CCB |
| Pancreatic | | | | |
| *AA* | C (67, 0.366) | C (no replication) | B (no obvious bias but no bias diagnostics) | CCB |
| *CA* | C (67, 0.366) | C (no association) | B (no obvious bias but no bias diagnostics) | CCB |
| *(AA+CA)* | C (67, 0.366) | C (no association) | B (no obvious bias but no bias diagnostics) | CCB |
| Nasopharyngeal | | | | |
| *AA* | C (14, 0.114) | C (no replication) | B (no obvious bias but no bias diagnostics) | CCB |
| *CA* | C (14, 0.114) | C (no replication) | B (no obvious bias but no bias diagnostics) | CCB |
| *(AA+CA)* | C (14, 0.114) | C (no replication) | B (no obvious bias but no bias diagnostics) | CCB |
| Endometrial | | | | |
| *AA* | C (24, 0.254) | C (no association) | B (no obvious bias but no bias diagnostics) | CCB |
| *CA* | C (24, 0.254) | C (no replication) | B (no obvious bias but no bias diagnostics) | CCB |
| *(AA+CA)* | C (24, 0.254) | C (no replication) | B (no obvious bias but no bias diagnostics) | CCB |
| Cervical | | | | |
| *AA* | C (32, 0.254) | C (no association) | B (no obvious bias but no bias diagnostics) | CCB |
| *CA* | C (32, 0.254) | C (no association) | C (nominal OR: 1.05) | CCC |
| *(AA+CA)* | C (32, 0.254) | C (no association) | B (no obvious bias but no bias diagnostics) | CCB |
| Ovarian | | | | |
| *AA* | C (11, 0.184) | C (no association) | B (no obvious bias but no bias diagnostics) | CCB |
| *CA* | C (11, 0.184) | C (no association) | C (nominal OR: 0.95) | CCC |
| *(AA+CA)* | C (11, 0.184) | C (no association) | C (nominal OR: 0.93) | CCC |
| Lung | | | | |
| *AA* | C (5, 0.076) | C (no replication) | B (no obvious bias but no bias diagnostics) | CCB |
| *CA* | C (5, 0.076) | C (no replication) | B (no obvious bias but no bias diagnostics) | CCB |
| *(AA+CA)* | C (5, 0.076) | C (no replication) | B (no obvious bias but no bias diagnostics) | CCB |
| Oral | | | | |
| *AA* | C (78, 0.409) | C (no replication) | B (no obvious bias but no bias diagnostics) | CCB |
| *CA* | C (78, 0.409) | C (no replication) | B (no obvious bias but no bias diagnostics) | CCB |
| *(AA+CA)* | C (78, 0.409) | C (no replication) | B (no obvious bias but no bias diagnostics) | CCB |
| Liver | | | | |
| *AA* | C (78, 0.409) | C (no association) | B (no obvious bias but no bias diagnostics) | CCB |
| *CA* | C (78, 0.409) | C (no association) | C (nominal OR: 0.88) | CCC |
| *(AA+CA)* | C (78, 0.409) | C (no association) | B (no obvious bias but no bias diagnostics) | CCB |
| Thyroid | | | | |
| *AA* | C (29, 0.278) | C (no association) | B (no obvious bias but no bias diagnostics) | CCB |
| *CA* | C (29, 0.278) | C (no replication) | B (no obvious bias but no bias diagnostics) | CCB |
| *(AA+CA)* | C (29, 0.278) | C (no replication) | B (no obvious bias but no bias diagnostics) | CCB |
| Lymphoma | | | | |
| *AA* | C (29, 0.278) | C (no association) | B (no obvious bias but no bias diagnostics) | CCB |
| *CA* | C (29, 0.278) | C (no association) | C (nominal OR: 0.94) | CCC |
| *(AA+CA)* | C (29, 0.278) | C (no association) | C (nominal OR: 091) | CCC |
